# Bronchial Carcinoma

**Published:** 1948

**Authors:** W. W. D. Thomson

**Affiliations:** Professor of Medicine, The Queen's University of Belfast


### The Bristol Medico-Chirurgical Journal

*"Scire est nescire, nisi id me*
*Scire alius sciret."*

AUTUMN, 1948.

### BRONCHIAL CARCINOMA

BY

W. W. D. Thomson, D.L., M.D., B.Sc., F.R.C.P.*

*Professor of Medicine, The Queen's University of Belfast.*

The first definite clinical description of cancer of the lung I can discover is given by Boerhaave (Zimmerman, 1782) with such dramatic skill that I cannot refrain from quoting some extracts therefrom. The Marquis of St. Auban was a strong and active man until a continual cough and persistent pain in his chest began to mar his previous splendid health. His breathing became difficult, he usually sat upon his bed, leaning forward with his elbows on his thighs. He could get no sleep night or day. In despair his physician sought the services of the great Dutch physician who put aside the tentative diagnosis of abscess of the lung owing to lack of constitutional symptoms, and inclined to the opinion that undue resistance of the thoracic walls caused the pain, dyspnœa and sense of suffocation. Ten months after the onset of symptoms the Marquis died. Boerhaave obtained permission to open the body which was, despite the victim's suffering, by no means emaciated. The whole left cavity of the chest was filled with a white substance, which had invaded the heart and compressed the bronchi. Boerhaave was stupefied at the sight of this singular phenomenon which he regarded as a steatoma and described the condition under the title "*Atrox Rarissimusque Morbus.*"

* From an address delivered to the Bristol Medico-Chirurgical Society on February 18th, 1948.

In 1912 Adler published his classical work, *Primary Malignant Growths of the Lungs and Bronchi*, and excited a fresh interest in the subject. Since then the great developments in radiology and bronchoscopy, in anæsthetics and thoracic surgery have given sufferers from this terrible and common disease a ray of hope, provided an early diagnosis be made. The urgency and importance of the problem is shown by the Registrar-General's latest statistical review, where we find that primary cancer of the lung in the male is exceeded in frequency only by cancer of the stomach, rectum and colon : and that 14 per cent. of men who die of cancer in the prime of life, between the ages of thirty-five and fifty-five, die from pulmonary carcinoma.

From the combined observations made by clinicians, bronchoscopists, radiologists and pathologists a fairly complete picture of cancer of the bronchus and its modes of behaviour can be obtained. The great majority of these growths have their origin in the main bronchus 1 or 2 cm. below the bifurcation of the trachea ; less frequently in bronchi of the second order. This is the hilar or central type. On the other hand the tumour may develop from one of the smaller bronchi producing a circumscribed tumour mass at a distance from the hilum—the peripheral or parenchymatous type. Kramer and Som (1936) have described the bronchoscopic appearance of a very early lesion in a patient who presented few pulmonary signs and in whom X-ray examination of the chest revealed no abnormality. The normal bronchial marking was distorted over a small area one centimetre in diameter where the rings seemed to be drawn close together and over which the mucosa presented a ruffled and wrinkled appearance. Biopsy of the overlying mucosa revealed an infiltrating squamous-celled carcinoma. From such a lesion growth may proceed by submucous infiltration around the bronchus. As growth proceeds more of the bronchial circumference is involved until a tubular stenosis is produced. On the other hand the growth may early break through the mucosa and protrude into the lumen appearing as a granular, friable and sessile tumour which quickly produces obstruction. Or again the primary growth, passing the cartilaginous barrier of the bronchial wall, may invade the lung tissue, being demonstrated on X-ray examination as a shadow at the hilum. These early stages are but passing phases, for soon occlusion of the bronchus follows and collapse ensues. Once collapse takes place the disease progresses rapidly.

*Clinical History*.—Let us pause to consider the possibility of diagnosis at this comparatively early stage, for in no malignant condition is early diagnosis more important. The great difficulty in early diagnosis lies in the paucity of symptoms. These are so unobtrusive that the patient does not seek medical advice, so commonplace that the physician does not suspect serious trouble.

Cough is the first symptom to appear : the onset of an unexplained cough should excite the suspicion of cancer of the lung when this symptom occurs in a patient of middle age hitherto free from respiratory ailments. Often the cough differs in no way from that found in ordinary catarrhal conditions of the bronchial tract. "Short", "dry", "something sticking that I cannot get up", are common descriptions. Its persistence and failure to respond to treatment are characteristic. Later, as the obstruction increases, the cough is apt to become paroxysmal, and at times resembles whooping-cough. An "asthmatoid wheeze" may attract the patient's attention (Jackson, 1930). The sputum at this stage, if present at all, is generally scanty and mucoid. If the sputum becomes streaked with blood the patient is fortunate, for medical advice will probably be sought. When streaking occurs it is apt to persist for days, but in this series hæmoptysis was recorded in less than 40 per cent. of the cases. In such an event pulmonary tuberculosis is generally considered, and the repeated failure to demonstrate tubercle bacilli and the absence of direct radiological evidence of disease in the chest, instead of exciting suspicion, is apt to engender a false feeling of security. The possibility of an early growth demonstrable by the bronchoscope is overlooked. Dyspnœa on exertion may be a comparatively early symptom. To sum up, the association of persistent cough, scanty sputum (especially if bloodstained) and respiratory discomfort should always in a male patient of thirty-five or over raise the suspicion of cancer of the lung and lead to a thorough investigation.

In the earlier stages of obstruction the lumen is simply narrowed : air can pass in and out, but in diminished amount. The "asthmatoid wheeze" can sometimes be found if listened for at the open mouth of the patient : this sound is rarely audible at the chest wall.

Later, obstruction has increased so that, though during inspiration dilatation of the invaded bronchus is sufficient to allow air to enter, on expiration it cannot escape. The result will be an emphysema in the corresponding lobe. It requires an expert to demonstrate convincingly the physical signs, and in the early stage clinical examination may yield completely negative results. But the suspicions roused by the patient's story must not be disregarded. Any sputum obtainable should be examined by the technique of Dudgeon and Wrigley (1935), who by means of a wet-film method were able to demonstrate particles of malignant growth. Out of 110 cases of primary cancer of the lung, diagnosis was made from the type of cell found in the sputum in sixty-eight.

*X-rays.*—In the very early stage no direct evidence of growth may be seen in plain X-ray film, but lipiodol may be of the greatest service. When the tumour grows into the lumen it produces a bronchogram showing a filling defect. The infiltrative type of growth produces a concentric filling defect with irregular borders. Bronchography is also of value in that it may reveal filling defects beyond the range of vision of the bronchoscope.

It has been mentioned that not infrequently the growth from the main bronchus may infiltrate the lung parenchyma before producing broncho-stenosis. In such an event X-ray examination discloses a shadow situated at the hilum. The border of this shadow may be well defined or may be ragged with strands of growth passing outwards along the branches of the bronchus. A lateral view should always be taken when such a hilar shadow is seen lest this may be a pseudo-hilar one. It is in the diagnosis of the hilar shadow that most radiological errors are made.

*Bronchoscopy.*—The bronchoscope offers the most decisive method of diagnosis, and may reveal a lesion too small to be demonstrated by any other method. Kramer and Som (1936) carried out a bronchoscopic examination in 300 cases of pulmonary carcinoma, and in 74 per cent. the diagnosis was confirmed by histological examination of biopsy tissue so obtained. By a combination of clinical, laboratory, radiological and bronchoscopic methods it is evident that, if suspected and sought for, many bronchial carcinomata situated in the neighbourhood of the hilum can be diagnosed in a relatively early stage.

*Late Stage.*—With the development of complete stenosis the disease enters on its terminal stage. The radiological signs of collapse appear, and the area supplied by the occluded bronchus is seen as an opaque shadow. The boundaries of the opaque area are concave (if viewed in a suitable projection) and the adjoining lobe or lobes have become emphysematous. In many cases at the site of the stenosis a rounded or irregular shadow fusing with the opaque area can be seen which represents the expanding tumour. The collapsed portion, its airless alveoli filled with exudate, forms a suitable nidus for inflammatory changes : hence pneumonic consolidation and abscess formation are frequent complications. The retention of septic material in the blocked bronchi and alterations in pleural pressure favour the progress of bronchiectasis. The pleura becomes involved in practically every case, an effusion may develop, adhesions may be produced or plaques of new growth stud the membrane. The growth may necrose and cavity formation occur. Hence many patients date the onset of their illness from symptoms referable to the pulmonary complications which follow in the wake of collapse. This was the most frequent type of onset found in this series, comprising 40 per cent. Chills, colds in the chest, "influenza", "pneumonia", pleural effusions with or without previous pain in the chest, were common clinical stories. It is surprising that the spitting of blood was recorded in only 37 per cent.

The persistence of the bleeding is a striking feature. The sputum often continues to be stained for weeks.

The usual clinical features found in this group are dullness on percussion with loss of breath sounds at one base. Often all signs of a pleural effusion are present, but the exploring needle fails to find fluid and may give the impression of entering a solid resisting mass. Occasionally the heart sounds can be heard distinctly over this dull area. The exploring needle passing through a pleural cavity, obliterated by adhesions, may enter an abscess or bronchiectatic cavity and withdrawing pus lead to an error in diagnosis. Or pleurisy with effusion, serous, hæmorrhagic or purulent, may be found. A latent and symptomless pleural effusion, especially if hæmorrhagic, is almost pathognomonic of bronchial carcinoma. Even when a pleural effusion masks the lesion stenosis of a bronchus can easily be demonstrated by tomography. When purulent fluid is withdrawn the diagnosis of empyema is a natural one. Operation was performed in nine cases in this series in which empyema or lung abscess had been diagnosed.

*Pain* occurred in 65 per cent. of my patients during some stage of their illness. In 15 per cent. it was the symptom for which they first sought medical advice. Pain is not a symptom in the early stage. In the hilar type pain is generally related to pleural involvement. Still later in the illness pain may be due to nerve compression or erosion of the bony framework of the thorax.

In 11 per cent. of this series the growth involved the bronchial glands to a greater extent than the lung : consequently pressure on structures in the mediastinum evoked the presenting symptom. In the course of the disease (though often late) nearly 50 per cent. develop pressure signs. The orthopnœa so often witnessed as a terminal event is found associated with mediastinal pressure narrowing the great air passages. Difficulty in breathing may at times simulate attacks of asthma.

A very important group causing great difficulty in diagnosis is the type whose initial symptoms are referable, not to the primary growth in the chest, but to metastases elsewhere. These constituted 10 per cent. of the series. Metastatic deposits in the brain arising from a latent cancer in the lung have been so often diagnosed as primary cerebral tumours that a routine X-ray of the chest should be carried out to avoid this source of error. The diagnosis is scarcely less puzzling when the presenting symptoms are caused by secondary deposits in bone. Metastases in the supraclavicular or axillary glands may be helpful in diagnosis. In one exceptional case an axillary gland became enlarged more than a year before any other evidence of the disease was present : but as a rule pulmonary growth is advanced before the superficial glands become palpable.

Symptoms which led to diagnosis in author's 130 cases may be summed as follows:

| | Per cent. |
|---|---|
| Early: cough, sputum (sometimes bloodstained), changes in respiration .. .. .. .. .. .. | 17 |
| Pulmonary complications following occlusion of bronchus | 40 |
| Pain .. .. .. .. .. .. .. .. | 15 |
| Pressure symptoms .. .. .. .. .. .. | 11 |
| Metastases .. .. .. .. .. .. .. | 10 |
| Others .. .. .. .. .. .. .. .. | 6 |

Other types of primary cancer of the lung are less frequently found. Of special importance are the tumours which are found separate from the hilum in the parenchyma of the lung. They arise from small bronchi. They are generally circumscribed and produce spherical, well demarcated growths in the lung. This peripheral type of tumour may remain latent for long periods. They are notoriously prone to give rise to early metastases in distant organs which may be the first indication of their presence. Mass radiography may demonstrate their unsuspected presence. The solitary rounded shadow found may be difficult to distinguish from an encysted interlobar empyema, from a hydatid or other cyst or from a benign neoplasm.

Among the Belfast series of cases a type in which the pleura was mainly involved was not uncommon, five cases being of this nature. Two of these were traced to a small adenocarcinoma originating in one of the lesser bronchi. In one of these an extension of the growth from the pleural mass invaded the vertebral column causing paraplegia. In three cases, however, the tumours were shown to be mixed-celled sarcomata. In each the growth was confined to one pleural sac and the bronchi were unaffected. The presenting symptom was pain which was accompanied by pleural friction and effusion.

*Differential Diagnosis.*—The difficulties introduced into the diagnosis of primary cancer of the lung by the pulmonary complications inherent in the disease or by metastases elsewhere in the body have been considered. Careful repeated bacteriological examination of the sputum and X-ray examination should exclude pulmonary tuberculosis. It must be remembered, however, that error is possible in the reverse direction, as the presence of tubercle bacilli in the sputum does not necessarily exclude cancer of the lung, the two conditions being occasionally co-existent.

The diagnosis between fibrosis and carcinoma of the lung is often very difficult. The patient's preceding history may assist, lipiodol injection will generally show patent bronchi and bronchiectatic cavities. Bronchoscopy will probably reveal a ragged granulomatous condition of the bronchi and quantities of pus may be seen exuding from their orifices.

In 6 per cent. of this series evidence of previous syphilis was obtained. Syphilitic sequelæ can closely simulate bronchial carcinoma. The differential diagnosis between aneurysm of the aorta and intrathoracic neoplasm is discussed in every text-book of medicine. In one case under my care the diagnosis was only settled by surgical exploration.

When Hodgkin's disease chiefly affects the mediastinal glands the simulation of primary cancer of the lung may be very close. Secondary growths in the lung from a latent primary focus may cause compression of a bronchus.

Considerable attention has been drawn in recent years to the question of benign tumours of the bronchi. By far the most important is the bronchial adenoma which generally arises in the primary or secondary bronchi. Their histological structure often suggests a malignant growth, but from their long clinical course and absence of metastases they are regarded clinically as benign. There are two distinct groups of symptoms—those due to the adenoma—cough and recurrent hæmoptysis, and those due to obstruction causing the secondary features of collapse and infection of the lung. Treatment is directed to prevent these sequelæ, and the prognosis is good only when diagnosis, which depends on bronchoscopy, is made early and followed by treatment.

### REFERENCES

Zimmerman (1782). *A Treatise on Experience.* London. Book iii, p. 232.

Adler, I. (1912). *Primary Malignant Growths of the Lungs and Bronchi.* New York.

Kramer, R., and Som, M. L. (1936). *Arch. Otolaryng.*, 23, 526.

Jackson, C. (1930). *J.A.M.A.*, 95, 640.

Dudgeon, L. S., and Wrigley, C. H. (1935). *J. Laryng. and Otol.*, 50, 752.

